# The relationship between countermovement jump force-time characteristics and 2,000-m rowing ergometer performance

**DOI:** 10.3389/fspor.2025.1549763

**Published:** 2025-03-31

**Authors:** Joseph M. DeLeo, Alex Wolf, Nicolas M. Philipp, Kathryn E. Ackerman, Andrew C. Fry

**Affiliations:** ^1^ Jayhawk Athletic Performance Laboratory-Wu Tsai Human Performance Alliance, University of Kansas, Lawrence, KS, United States; ^2^ Female Athlete Program-Wu Tsai Human Performance Alliance, Division of Sports Medicine, Boston Children’s Hospital, Boston, MA, United States; ^3^ Strength & Conditioning Academy Ltd., London, United Kingdom

**Keywords:** countermovement jump (CMJ), rowing, force-time characteristics, impulse, 2,000-m

## Abstract

**Background:**

Rowing is a strength-endurance sport. The Olympic race distance of 2,000-m (2k) requires extensive aerobic and anaerobic energy system contributions, along with sustained high force output.

**Aim(s):**

The primary aim of this study was to evaluate the relationship between rowing ergometer (erg) performance and the force-time curve characteristics of a counter-movement jump (CMJ), and to determine if rowing-related low back pain (LBP) affected these relationships.

**Methods:**

Athletes completed a 2k time trial at the 2023 USRowing Atlantic City Indoor National Championships as well as a CMJ on force plates. Data from the 2k (*n* = 27) time trial was analyzed to determine significant relationships between CMJ force-time characteristics and 2k rowing ergometer performance. Partial correlations were used to determine the effects rowing-related LBP has on the relationship between 2k erg and CMJ force-time curve characteristics.

**Results:**

Positive Take-off Impulse had the strongest correlation with 2k erg performance (*r* = 0.71, *p* < 0.001). Jump height was not significantly related to 2k erg performance (*r* = −0.13, *p* = 0.518). Current LBP changed the relationship for Concentric Mean Force (*r* = 0.74, *p* < 0.001).

**Conclusion:**

Positive Take-off Impulse has a strong, positive relationship to 2k erg performance. CMJ variables related to impulse and force should be monitored longitudinally to see if changes in these variables coincide with improved 2k rowing erg performance and the assessment of neuromuscular fatigue. Coaches can utilize these findings to individualize strength and power training for potential 2k erg performance improvements.

## Introduction

Rowing has been classified as a strength-endurance sport (1, 2) due to the extensive aerobic and anaerobic demands of racing that have on-water world best times lasting between 5:18.68 and 7:23.36, depending on boat classification (3). The energy system contribution to racing has been determined to be approximately 70%–77% aerobic and 23%–30% anaerobic (4, 5). Ingham et al. had 41 World Rowing Championships finalists perform the following series of tests to identify the physiological determinants of 2k rowing ergometer performance: a 2k ergometer (erg) time trial, a discontinuous incremental test to establish the blood lactate threshold, a seven-stroke test (two building strokes, five maximal) to determine maximal force (F_max_), maximal power (W_max_), and stroke length. W_VO2max_ (power at maximal oxygen consumption) and W_max_ were the strongest single correlates to 2k erg performance (6). Further analysis determined that 98% of 2k erg variance could be explained by W_VO2max_, VO_2LT, LSS_ (oxygen consumption at lactate threshold determined by least sum of squares), W_max_, and W_4mmol^−1^_ (power at blood lactate concentration of 4 mmol/L^−1^) (6). Ingham's study highlighted that rowers require significant development of both aerobic (W_VO2max_) and anaerobic (W_max_) energy systems for 2k performance. However, all of these tests were conducted on a rowing ergometer. The inclusion of strength and neuromuscular tests that assess and monitor anaerobic characteristics are valuable to the development of rowers to understand the specific anaerobic qualities that may contribute to performance.

Strength has been defined as the ability of the body to produce force to overcome inertia or load (7) whereas maximal force (F_max_) is defined as the maximum amount of force a muscle or muscle group can generate (7, 8). Athletes are often required to exert maximal forces, externally, in several ways: to overcome gravity (e.g., sprinting, high jump), to move a competitor's body mass (e.g., wrestling), to use an instrument (e.g., hockey stick, baseball bat, soccer), and to propel a piece of equipment or an implement (e.g., cycling, rowing, javelin) (9). Therefore, an athletes' F_max_ is important to develop for sport performance, as well as the rate of force development (RFD), defined as the greatest magnitude of force an athlete can produce in a time constraint (8, 10). Many endurance sports have a strong correlation between a high RFD and sport performance. For example, running (11) and cycling (12, 13) have utilized the Isometric Mid-Thigh Pull (IMTP) for RFD measurement whereas in on-water rowing it's traditionally been measured at the pin of the oarlock (14, 15). However, one of the practical limitations of the IMTP is that it takes significant time to set-up and execute. This makes the IMTP a challenging test to implement in sports such as rowing that have teams of 40 + athletes (16, 17).

A recent study by Ledergerber et al. investigated the contributions of different strength determinants for different phases (start, middle, end) of 2k erg performance in 14 adolescent rowers (18). Ledergerber found that the RFD of isometric leg press over 150 and 350 milliseconds (ms) was significantly correlated (*r* = 0.671–0.918, *p* < 0.05) to the start, middle, end and total 2k race distance (18). A final key finding was that the single greatest predictor for 2k erg performance was attributed to absolute VO2_max_, maximal isokinetic trunk flexion, and sex, explaining 97.5% of the variance (*p* < 0.001). This provides evidence that reinforces the importance of developing aerobic metabolism to improve VO2_max_ and F_max_ transmission through the legs and trunk.

Strength-endurance athletes, such as rowers, benefit from strength training through improved economy and muscular power (19), contributing to ∼30% of the anaerobic energy supply (4). Reviewing data from Steinacker, for each stroke during on-water competition, elite male rowers produce peak forces of 1,000–1,500 Newtons (N) at the start, 500–700 N during the body of the race, and 600–700 N for the final sprint of a 2k race (20). Analyzing data from each stroke of 47 2k races during two regattas at the Sydney International Rowing Center, Holt et al. found that the mean force across 2k was 261 N in the Men's Single (M1x), 199 N in the Women's Single (W1x), 503 N in the Men's Pair (M2-), and 367 N in the Women's Pair (W2-) (14). Furthermore, the time to peak force from the catch (blade entering the water at the start of each oar stroke) ranged from 0.36 to 0.43 s across these boat classes (14). Taken together, these two studies highlight the importance of F_max_ (20) and RFD (14) for on-water rowing performance. To further tie together on-water rowing performance and on-land strength determinants, Ledergerber found that the RFD of the IMTP over 300 ms was significantly correlated (*r* = 0.769–0.903, *p* < 0.05) to the start, middle, end and total 2k race distance, but not at 150 ms (*r* = 0.302, to −0.413, *p* > 0.05) (18). This shows that regardless of environment (on-water or on-land) rowers have a longer RFD that relates to rowing performance (14, 18).

The countermovement jump (CMJ) is a vertical jump test performed by having an athlete start in a standing upright position, hands on hips, before making a downward movement with a triple flexion of the hips, knees, and ankles, and then aggressively extending the hips, knees, and ankles to jump off the floor as high as possible (21). The CMJ requires a coordinated flexion of the ankles, knees, and hips; followed by a rapid full extension of each joint to accelerate off the floor; then concluding with a landing back on the ground. The CMJ has been used across multiple sports as a proxy measure of muscle force and power (22, 23). Dos' Santos et al. conducted a study investigating IMTP against CMJ, squat jump, and 1 rep maximum power clean in 43 (rowing, soccer, motocross, and hockey) athletes. They found that only force at 250 ms in the IMTP had a significant, yet moderate correlation (Spearman = 0.346, *p* = 0.016) with CMJ jump height (24). Boullosa et al. investigated the force-time characteristics of different athletic populations and the relationships between them. The study included three groups: endurance (eight runners, six triathletes), 12 sprinters, and 12 fire fighters (controls). The main finding was that the CMJ force-time characteristics were dependent on training background. In terms of absolute values, the sprinters exhibited greater jump height, peak power, normalized vertical stiffness and RFD compared to the endurance and fire fighters (25). However, the strength of the correlations for many of the force-time variables were higher in the endurance group. For example, when examining the relationship between vertical stiffness and the ratio between peak RFD and its time of occurrence they found the endurance group (*r* = 0.920, *p* < 0.01) had a significantly stronger relationship compared to the sprinters (*r* = 0.721, *p* < 0.01) (25). This indicates that the athletes who could producer higher levels of vertical stiffness at the end of the eccentric phase were able to translate that to earlier and higher RFD values (25). This indicates that different athletic populations not only achieve different levels of absolute force but ***how they produce*** this force is distinct to their sporting background.

The CMJ has been extensively used within elite rowing populations for over a decade with key force-time curve characteristics being used to measure both improvements with strength training as well as demonstrate the strong relationship between leg strength and power in 2k erg, a key selection metric for crews (8, 26, 27). Podstawski et al. explored the relationship between CMJ and 2k erg in 200 rowers (female: *n* = 70, male: *n* = 130) and found, in both sexes, that 2k erg time was significantly shortened (*p* < .001) with an increase in peak power (*r* = −.98 and −.99), relative peak power (*r* = −.77 and −.76), and F_max_ (*r* = −.59 and −.52) during the CMJ (28). Interestingly, male rowers displayed a significantly shorter 2k erg with an increase in jump height (*r* = −.36, *p* < .001) (28). The rowers in this study were 15–22 years old, which limits the ability to generalize findings to other populations. Further research is warranted to establish if a relationship exists between CMJ performance and 2k rowing erg performance within the sport of rowing and between both sexes. Therefore, the purpose of this study was to evaluate the relationship between 2k erg and CMJ, and associated force-time curve characteristics related to force, power, and impulse. A secondary aim was to determine if sex and self-reported rowing-related low back pain (LBP) affected the relationship between 2k erg and CMJ.

## Materials and methods

The study was completed at the 2023 USRowing Atlantic City Indoor National Championships (2023 USACINC) that were held on February 4th–5th, 2023 in Atlantic City, New Jersey, USA.

### Participants

A convenience sample of participants who were competing at USACINC were recruited to participate in this study. The inclusion criteria included participants who were ≥18–49 years of age and registered to compete in the 2023 USACINC. Exclusion criteria included 2023 USACINC participants who were <18 years of age, or in the PR3 Down Syndrome (DS) and PR3 Intellectual Impairment (II) competition categories due to concerns around safely landing after performing a CMJ; all other para categories were included. Informed consent was obtained electronically from each participant via an iPad using a secure link from Qualtrics (Seattle, WA). Research approval was obtained from the University of Kansas Institutional Review Board (STUDY00149616).

#### Indoor rowing tests

The 2023 USACINC included events ranging from juniors to masters rowers across multiple distances including max watts testing, 500 m, 2k, four-person team relay across 2k, and triathlon (RowErg, BikeErg, and SkiErg). The data included in our study was participants' publicly available 2k time trial data. All testing was performed on a RowErg (Model D; Concept II, Inc., Morrisville, VT, USA). Per USRowing rules, rowers were allowed to set the drag factor to their desired setting (29).

### Procedure

The research team were initially positioned in an exhibitor's booth by the competition area and then moved to the warm-up area to increase the recruitment of participants. USRowing circulated an email to all indoor rowing competitors the weekend of the event, informing them of the research study, where the study was being conducted, and the testing involved.

### Demographic and training history survey

After informed consent, participants completed a survey through Qualtrics (Provo, UT). The survey questions included the participant's name, age, competition category at the 2023 USACINC, rowing experience, and history of self-reported rowing-related low back pain. The research team was then able to link participants' survey responses, CMJ tests, and their 2k erg at the 2023 USACINC via the publicly available results on Time-Team Regatta Systems (Amersfoot, Netherlands).

### Countermovement jump (CMJ)

Dual uni-dimensional force plates (VALD ForceDecks, Brisbane, Queensland, Australia) system were used to capture the force-time characteristics of the CMJ. VALD ForceDecks' CMJ metrics of Take Off Peak Force (N), Positive Take-off Impulse (N • s), and Jump Height (Imp-Mom) (cm) have been found to have good to excellent concurrent validity with force plates embedded in the floor (AMTI, MA, United States); only showing relative differences of 0%, 1%, and 5%, respectively (30). This system is comprised of a bilateral, one-dimensional set of force plates, with a sampling frequency of 1,000 Hz. Members of the research team calibrated the force plates according to manufacturer specifications, by zeroing them out, ensuring no external mass was touching them, prior to the participant stepped onto them to complete CMJ testing (31). Participants' body mass was auto calculated by the force plates prior to their first jump. Test data was stored on the VALD Hub, a cloud-based analytics software.

Prior to CMJ testing, participants completed a familiarization of the jump protocol consisting of 3–5 jumps, with coaching instruction from the researchers. Participants completed three CMJs with each CMJ separated by 10–15 s. Participants executed the CMJ as described in the introduction and were encouraged to jump as high and as fast as possible. To increase study participation, rowers in this study had the opportunity to complete the CMJ at their convenience during the 2023 USACINC, including pre or post competition. The six phases of a CMJ are the weighing phase (32), start of movement, eccentric phase, concentric phase, take-off phase, and landing phase (33). The CMJ variables of interest were concentric mean force (N), concentric mean power (W), concentric peak force (N), jump height (impulse-momentum) (cm), peak power (W), countermovement depth (cm), and positive take-off impulse (N • s). Positive take-off impulse as defined here is the same as the vertical jump impulse that is used for the impulse-momentum calculation for jump height. Additionally, the relative temporal variables of concentric mean force/BM (N/kg), concentric mean power/BM (W/kg), and eccentric mean deceleration force (N) were evaluated. The time of the stretch-shortening cycle (T_SSC_) was calculated by summing the concentric and eccentric durations as described elsewhere (34). Table 1 provides definitions of CMJ phases and variables.

**Table 1:** CMJ phases & variables.

Variable	Definition
Weighing phase^a^	Silent period where the athlete stands still and body mass is calculated
Start of movement^b^	Moment where force deviates from steady-state
Eccentric phase^b^	Begins with “Start of Movement” and ends at moment of zero velocity
Concentric phase^b^	Begins at moment of zero velocity and ends at take-off
Take-off phase^b^	Where vertical force drops below 20 N after start of movement
Landing phase^b^	Where vertical force rises above 20 N after take-off
Concentric mean force (N)^b^	Average vertical force during concentric phase
Concentric mean power (W)^b^	Average power during the concentric phase
Jump height (imp-mom) (cm)^b^	Jump height calculated from the velocity of the COM at the instant of Take-off and body mass
Peak power (W)^b^	Maximum power during the concentric phase
Countermovement depth (cm)^b^	Maximum displacement between start of movement to take-off
Positive take-off impulse (N • s)^b^	Net impulse during the entire repetition (eccentric and concentric phases combined)

^a^ Definition from McMahon et al. (32).

^b^ Definition from VALD ForceDecks technical glossary (31).

#### Statistical analysis

Statistical analyses were performed in Microsoft Excel (Redmond, WA) and Jamovi (Sydney, Australia) (35). Data visualizations were created in R Studio (Version 2024.12.0 + 467) (36) using the following packages: “ggplot2”, “corrplot”, and “cowplot”. Categorical variables were reported as frequencies and percentages. Continuous variables were evaluated descriptively using means and standard deviations or median [interquartile range (IQR)] for non-normally distributed data. All variables of interest were tested for normality using the Shapiro–Wilk test. Values were rounded up to the nearest hundredth. A Bonferroni correction was applied, setting statistical significance to *p* ≤ 0.007 (37); this was implemented to avoid a potential Type I Error and to account for the seven CMJ variables being analyzed from the same data set (38). The strength of the Pearson r correlation was defined as follows as 0 = zero, ±0.1–0.3 = weak, ±0.4–0.6 = moderate, ±0.7–0.9 = strong, and 1 = perfect (39).

Our analysis focused on the 2k erg time trial. The finish times for each participant (2k erg time) was converted to watts using the Concept2 pace calculator available on their website (40). The conversion of the participant's finish time to watts allowed a direct comparison of the mean power produced over the 2k erg time trial with the power and force-time curve characteristics performed during the CMJ. A correlation matrix was run between 2k watts (2k_watts_) and the CMJ variables of interest. The selected CMJ variables were related to force, power, and impulse as these are some of the most common force-time variables used in rowing stroke analysis (14, 41–44). Partial correlations were conducted, controlling for previous and current self-reported rowing-related low back pain. 95% Confidence Intervals (95% CI) are provided for all correlational analyses.

## Results

Table 2 provides a breakdown of the characteristics of participants. A total of 30 rowers (male = 27, female = 3) participated in this study; a single male participant competed in two events. Unfortunately, due to the small sample of female participants, we were unable to control for sex in our partial correlation analysis and excluded all female participants from our final data analyses, thus our final total was 27 male rowers. The rowers' competing at the 2023 USACINC had 2k erg times ranging from 6:07.3 to 7:58.2 and their ages were between 18 and 46 years old. The rowers' on-water and indoor rowing experience ranged from 0 to 22 years indicating various training ages specific to rowing and reflective of their 2k erg performances.

**Table 2:** Participant characteristics $\bar{X}$ ± SD.

Variable	All
N	27
Age (years)^a^	20.0 ± 4.5
Height (cm)	183.2 ± 8.7
Weight (kg)	86.7 ± 15.0
On-water rowing experience (years)^a^	4.0 ± 4.0
Indoor rowing experience (years)^a^	4.0 ± 3.5
Previously had rowing-related low back pain (%)	16 (59.3)
Currently have rowing-related low back pain (%)	6 (22.2)
2,000-m mean watts	333.7 ± 79.7
CMJ variables	–
Concentric mean force (N)	1,461 ± 265.3
Concentric mean power (W)	1,965 ± 405.4
Concentric peak force (N)	1,842 ± 319.3
Jump height (Imp-Mom) (cm)	30.8 ± 5.5
Peak power (W)	3,872 ± 678.2
Countermovement depth (cm)	−37.0 ± 9.0
Positive take-off impulse (N • s)	304.8 ± 56.6

^a^ Denotes non-parametric variable and is reported as median and interquartile range (IQR).

**Figure 1:**
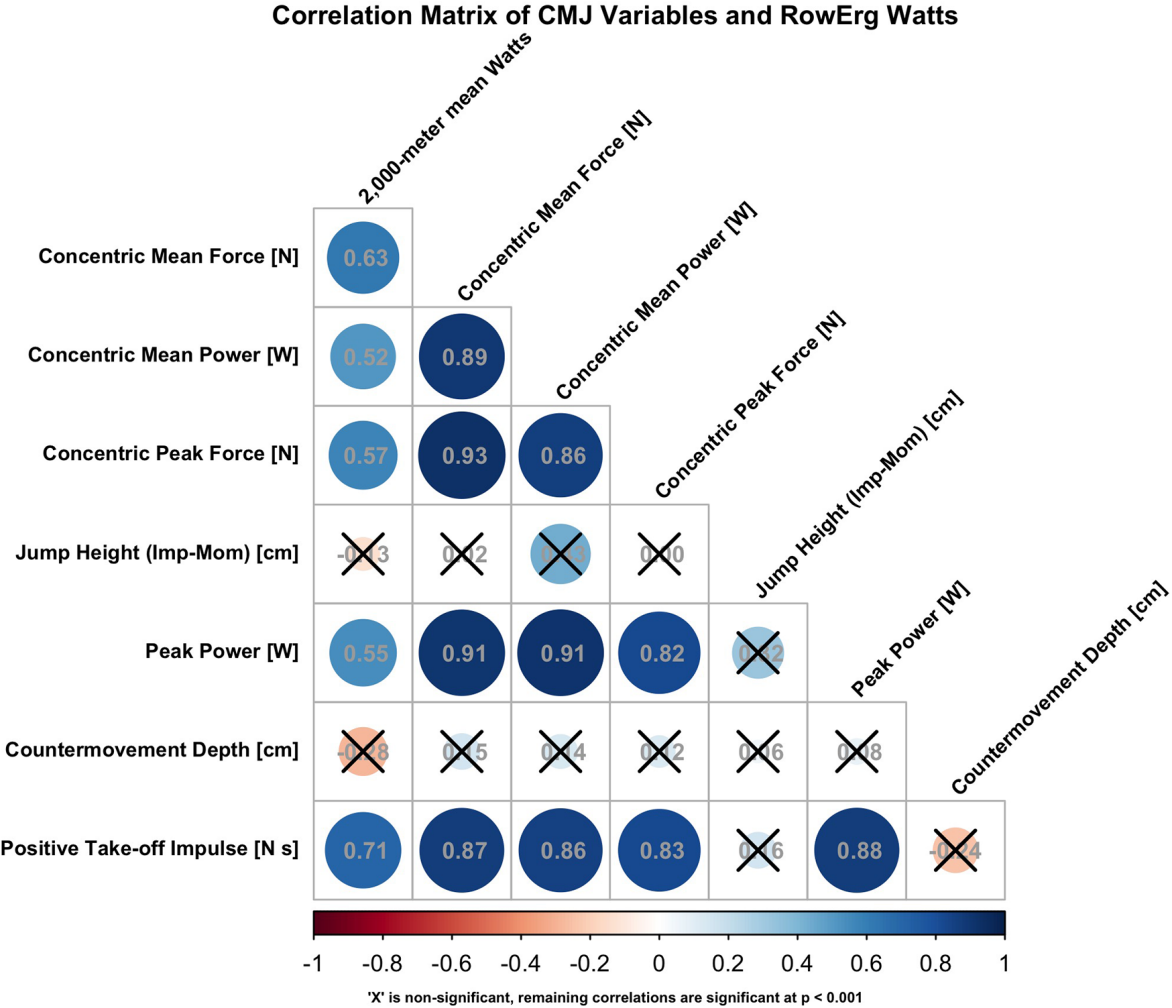
Heatmap Correlation Matrix.

Positive Take-off Impulse (N • s), was the only CMJ variable that demonstrated a strong positive relationship, and a statistically significant correlation across 2k_watts_ (*r* = 0.71, *p* < 0.001). The CMJ variables concentric mean force (N; *r* = 0.63, *p* < 0.001), concentric mean power (W; *r* = 0.52, *p* = 0.006), concentric peak force (N; *r* = 0.57, *p* = 0.002), and peak power (W; *r* = 0.55, *p* = 0.003) all displayed moderate positive, statistically significant correlations with 2k_watts_. This suggests that higher levels of force produced over a long period of time occur during the CMJ and potentially may be important for rowing performance. Jump height (impulse momentum) (cm; *r* = −0.13, *p* = 0.518) and countermovement depth (cm; *r* = −0.28, *p* = 0.156) had weak, negative relationships to 2k_watts_. A heatmap correlation matrix (Figure 1) is provided to visualize the strength of relationships between CMJ variables and 2k_watts_. The complete summary of correlations between CMJ variables and 2k_watts_, are found in Table 3 and presented in Figure 1.

**Table 3:** Correlations Between Rowing Ergometer Performance (Watts) and CMJ Variables.

Variable	2,000-m watts
Concentric mean force (N)*	–
Pearson *r*	0.63
*p*-value	<0.001
95% CI (lower to upper)	0.33–0.81
Concentric mean power (W)*	–
Pearson *r*	0.52
*p*-value	0.006
95% CI (lower to upper)	0.17–0.75
Concentric peak force (N)*	–
Pearson *r*	0.57
*p*-value	0.002
95% CI (lower to upper)	0.24–0.78
Jump height (Imp-Mom) (cm)	–
Pearson *r*	−0.13
*p*-value	0.518
95% CI (lower to upper)	−0.48–0.26
Peak power (W)*	–
Pearson *r*	0.55
*p*-value	0.003
95% CI (lower to upper)	0.21–0.77
Countermovement depth (cm)	–
Pearson *r*	−0.28
*p*-value	0.156
95% CI (lower to upper)	−0.60–0.1110
Positive take-off impulse (N • s)*	–
Pearson *r*	**0.71**
*p*-value	<0.001
95% CI (lower to upper)	0.44–0.86

* Significance at *p* < 0.007.

**Bold** values indicate a strong relationship.

Correlation coefficients are classified as weak, *r* = 0.1–0.3, moderate, *r* = 0.4–0.6, strong, *r* = 0.7–0.9, perfect, *r* = 1 (39).

When controlling for self-reported rowing-related LBP, positive take-off impulse was the only variable that displayed a strong, positive and significant correlation for both previous and current LBP (N • s; *r* = 0.75, *p* < 0.001; *r* = 0.76, *p* < 0.001, respectively) and 2k_watts_. Concentric mean force was the only other variable that showed a strong, positive relationship (N; *r* = 0.74, *p* < 0.001) to current rowing-related LBP. This suggests that self-reported rowing-related LBP regardless of status (previous or current) impacts performance over the 2k race distance. The complete summary of the partial correlations between rowing-related LBP for 2k_watts_ are found in Table 4.

**Table 4:** Partial Correlations for rowing ergometer performance (watts) after controlling for previous and current rowing-related LBP.

Variable	2,000-m (previous LBP)	2,000-m (current LBP)
Concentric mean force (N)*	–	–
Pearson *r*	0.66	**0.74**
*p*-value	<0.001	<0.001
95% CI (lower to upper)	0.37–0.83	0.49–0.87
Concentric mean power (W)*	–	–
Pearson *r*	0.59	0.66
*p*-value	0.001	<0.001
95% CI (lower to upper)	0.27–0.79	0.38–0.83
Concentric peak force (N)*	**–**	**–**
Pearson *r*	0.63	0.66
*p*-value	<0.001	<0.001
95% CI (lower to upper)	0.32–0.81	0.36–0.83
Jump height (Imp-Mom) (cm)	**–**	**–**
Pearson *r*	−0.08	−0.05
*p*-value	0.671	0.783
95% CI (lower to upper)	−0.45–0.31	−0.43–0.33
Peak Power (W)*	**–**	**–**
Pearson *r*	0.57	0.64
*p*-value	0.002	<0.001
95% CI (lower to upper)	0.24**–**0.78	0.34**–**0.82
Countermovement depth (cm)	–	–
Pearson *r*	−0.25	−0.18
*p*-value	0.201	0.367
95% CI (lower to upper)	−0.58–0.14	−0.53–0.21
Positive take-off impulse (N • s)*	**–**	**–**
Pearson *r*	**0.75**	**0.76**
*p*-value	<0.001	<0.001
95% CI (lower to upper)	0.52**–**0.88	0.54**–**0.89

**Bold** values indicate a strong relationship.

Correlation coefficients are classified as weak, *r* = 0.1–0.3, moderate, *r* = 0.4–0.6, strong, *r* = 0.7–0.9, perfect, *r* = 1 (39).

* Significance at *p* < 0.007.

## Discussion

The primary aim of this study was to evaluate the relationship between 2k erg and CMJ performance and its associated force-time curve characteristics related to force, power, and impulse. This is the first study to identify that the CMJ variable of positive take-off impulse was related to 2k erg performance. This is noteworthy because impulse is a direct reflection of an athlete's power, or work, achieved during the propulsive phase of the rowing stroke (42, 45). Force-time curve analysis of the rowing stroke is often used to assess several physical and biomechanical variables. This includes training intensity, timing, and rhythm between rowers in team boats, time to peak force, power, and impulse (14, 46). Impulse, defined as the area under the force-time curve (F x t), provides insight into the propulsive force generation of the rowing athlete (47). International level rowers have been shown to produce a larger impulse than national level rowers (48). Effective strength training focusing on F_max_ and power characteristics to support rowing performance may allow endurance athletes to utilize a lower percentage of their F_max_ capacity when training at low-intensity and to increase their velocity or power at maximal intensity (19). When evaluating rowing performance, power, defined as force multiplied by velocity (49), becomes an even more important physical quality and highlights the underlying need for high levels of strength and F_max_ development. Recent on-water research continues to support that early peak force and RFD translate to faster boat velocities as well as a larger impulse in the force-time curve (14, 15). However, it still needs to be determined if rowers with the greatest impulse during a CMJ also produce the greatest impulse during on-water or ergometer rowing. This is an area that needs to be explored in future research as this can have value purely beyond performance enhancement but also potentially utilized for talent identification in developing rowing athletes.

**Figure 2:**
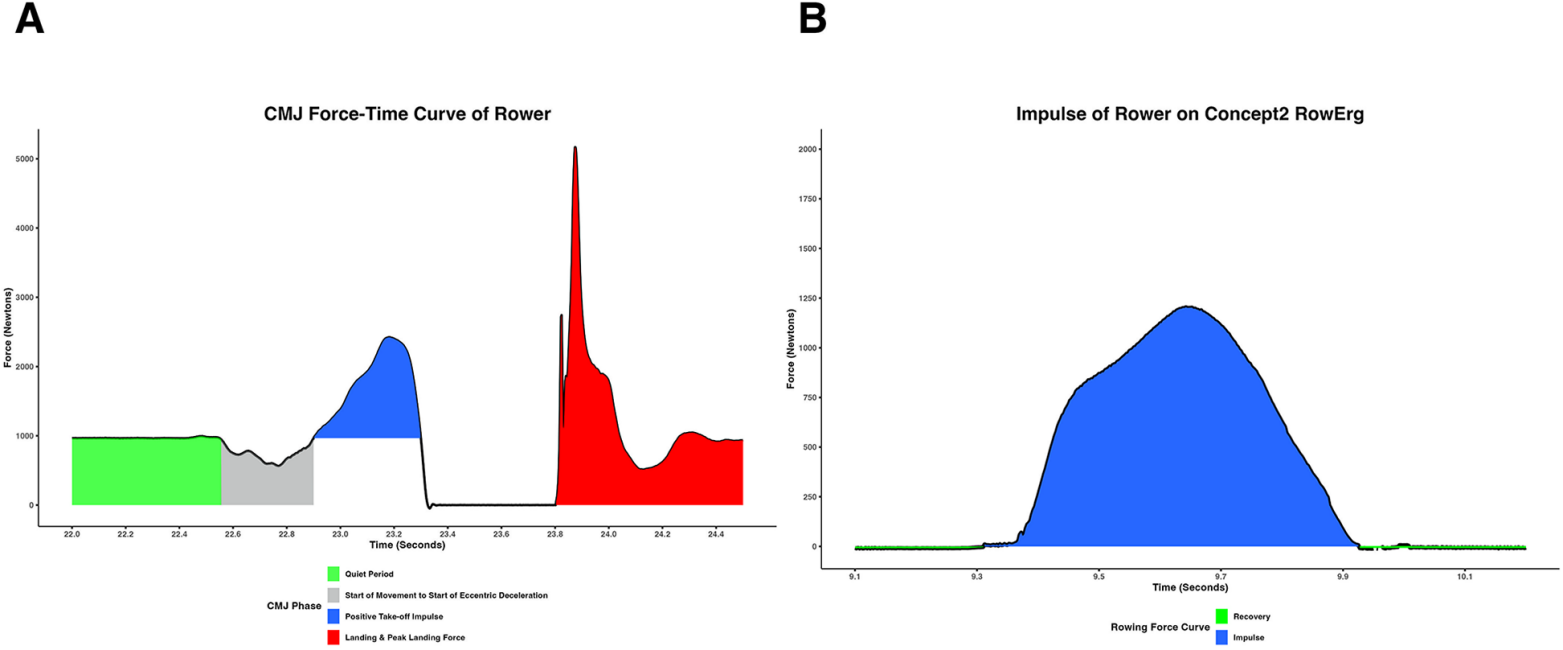
Comparison of CMJ **(A)** and rowing **(B)** force-time curves.

Second, positive take-off impulse demonstrated the strongest relationship to 2k_watts_ compared to concentric impulse (N • s; 2k_watts_ *r* = 0.57, *p* = 0.002). This indicates that the sum of the eccentric and concentric impulse phases of the CMJ have a stronger relationship to rowing ergometer performance than concentric impulse alone. This is insightful because the drive (propulsive) phase of the rowing stroke has been considered a concentric dominant movement. However, recent research by Held and colleagues investigating on-water rowing, CMJ, and drop jumps has provided evidence that rowing involves a slow stretch-shortening cycle (SSC) of the vastus lateralis, vastus medialis, and gastrocnemius muscles (34) and that the SSC contributes mainly to high-intensity rowing (50). The SSC involves a pre-activation of the muscle before the eccentric phase, a short and fast eccentric phase, and finally a short delay before the concentric phase (51). The SSC increases the rate and magnitude of the stretch on muscles resulting in a higher force output (51) and has been classified as short (fast) (<0.250 ms) or long (slow) (>0.250 ms) (52). Held et al. found the removal of the eccentric portion of the flexion-extension cycle (FEC) within the rowing stroke, through the use of micro-pausing rowing, significantly reduced the SSC and reduced the onset and amplitude of EMG activity when compared to traditional FEC rowing (50). In a separate study, Held et al. found that 10 elite male rowers' time of the SSC (T_SSC_) was 731 ± 217 ms during the CMJ (34). The male rowers in our study displayed a mean T_SSC_ of 1,049 ± 215.4 ms. Taken together, these findings suggest additional evidence of a slow SSC occurring during the CMJ in this cohort of rowing athletes. The differences in our T_SSC_ may be attributed to the comparison of elite rowers who were all World Championship medalists against university, masters, and recreational rowers (34). Additionally, all of the elite rowers in Held's study had an average age of 22.8 ± 3.1 whereas our cohorts were not normally distributed (20.0 ± 4.5) including six participants over the age of 25 (ages 27–46). Future research should investigate long SSC exercises within the strength and conditioning program to determine if this outcome-based approach results in an improvement in rowing performance. For example, potential exercises may include the squat jump (27) or a weighted countermovement jump and/or modified plyometric exercises that focus on long and not short SSC. Figure 2 shows a comparison of CMJ and rowing force-time curves.

There are several noteworthy takeaways when evaluating our findings compared to research currently available in the literature. First, we found that jump height did not show a relationship to 2k erg performance which contradicts the findings of Podstawski et al. (28). This may be due to the fact that our jump height variable was calculated based on the impulse-momentum relationship which has been found to be the most reliable method (53), whereas Podstawski et al. directly calculated jump height from ground reaction forces without explicitly integrating force over time to determine take off velocity (28), which may account for the differences in results.

Additionally, the majority of Podstawski's cohort were junior male and female rowers at ages 15–16 (36 female, 55 male), 17–18 (26 female, 52 male), and 18+ (8 female, 23 male) which may affect the interpretation of results (28). In a separate study by Alfoldi, junior male rowers showed a lower jump height, peak power (W), and relative peak power (RPP W/kg) compared to their older peers (54). This indicates that simply the differences in chronological and training age may contribute to the differences in results between our studies. However, what is significantly different is our results for RPP compared to Alfoldi and colleagues. In our study, we found RPP to be 44.93 ± 5.3 W/kg compared to 3.76 W/kg (age 15–16), 4.42 W/kg (ages 17–18), and 4.59 W/kg (ages 19–22) (54).

Metikos et al. sought to determine the relationship between relative peak power (W/kg) from a CMJ and peak output during a 6-stroke peak power test on a Concept II rowing ergometer instrumented with a strain gauge, at three different drag factors: 90, 125, and 200 (55). A subset of their rowers (male = 15, female = 9) showed correlations of 0.76, 0.76, and 0.78 for each resistance level, respectively (55). This is additional evidence of the relationship between the CMJ force-time characteristics and rowing ergometer power output at a shorter time/distance. The 6-stroke peak power test would primarily be utilizing the phosphagen creatine energy system, whereas our study showed strong relationships in the CMJ between the aerobic (2k) energy system.

Rowers with previous and current rowing-related LBP showed a nearly identical strength of relationship to 2k_watts_ (*r* = 0.75, *p* < 0.001 vs. *r* = 0.76, *p* < 0.001). Interestingly, concentric mean force (N) had a change in the strength of relationship with previous LBP having a moderate positive relationship (*r* = 0.66, *p* < 0.001), while current LBP had a strong, positive relationship (*r* = 0.74, *p* < 0.001). This indicates that rowers who currently had LBP may have their rowing performance more negatively impacted than those with previous LBP. In fact, recent research by Martinez-Valdez et al. has shown that rowers who recently had rowing-related LBP exhibited altered muscle activation in the erector spinae during incremental rowing in a 7 × 4′ graded exercise test (56). Both the magnitude and distribution of erector spinae muscle activity was higher in rowers with rowing-related LBP. This alteration in muscle recruitment, firing, and activity may alter other motor patterns such as the rate, timing and muscle contraction in the CMJ. Thus, future research should be conducted to determine how it may impact training and athletic movements beyond rowing and jumping.

The CMJ has successfully been used to monitor neuromuscular fatigue in individuals (57, 58), team sport athletes (59, 60), and the military (61, 62), future research should investigate if changes in the force-time curve for both the CMJ and rowing stroke (on-water or rowing ergometer) can be used to determine acute and chronic neuromuscular fatigue, as well as to monitor rowers' performance across training cycles. Recent research by Everett et al. evaluated a loaded countermovement jump in 20 elite male rowers within and between pre-competition and competition mesocycles (63). The rowers were categorized into two groups: ones that had attained benchmarks of >1.7× bodyweight back squat, >1.1× bodyweight power clean, and >1.3× bodyweight bench press and those that did not. Rowers who did not achieve these strength standards had a 3.2% decrease in mean power in the loaded countermovement jump vs. 2.3% in the group that achieved those benchmarks (63). However, the loaded countermovement jumps were only completed in the first and last week of each mesocycle. Therefore, future research should aim to monitor changes in the CMJ on a weekly basis to identify more subtle changes in neuromuscular status. Additionally, the CMJ variables we identified in our study that had the strongest relationship to 2kerg performance, positive take-off impulse, concentric mean force, and peak power should be monitored to determine if they are more sensitive to these changes in neuromuscular status.

Finally, the relationship between 2k performance and body mass is well established. As body mass increases rowing performance improves (6, 64). This is supported in our study as well where we found that body mass was positively, moderately (*r* = 0.65, *p* < 0.001) to 2k erg performance. Furthermore, rowers who have higher levels of fat free mass (65) and muscle mass (66) have better 2k erg performance whereas increased body fat percentage negatively impacts 2k erg performance (6). However, when we look at the relative temporal CMJ variables in our study we found weak and non-significant relationships. For example, concentric mean force/BM (N/kg) (*r* = −0.0094, *p* = 0.963) and concentric mean power/BM (W/kg) (*r* = −0.05, *p* = 0.808) had a nearly zero relationship to 2k erg performance. This is a critical insight because this indicates absolute values of force, power, and impulse are potentially more important than relative values. Second, when we look at eccentric mean deceleration force (N) (*r* = 0.69, *p* < 0.001) this had a positive, nearly strong relationship to 2k erg performance. This indicates that a rowers' ability to turn around and apply concentric force is highly related to 2k erg performance—which is exactly what a rower needs to execute during every rowing stroke as they complete the recovery and begin the drive phase of the rowing stroke. Therefore, rowers who can control their body mass and change directions while applying high levels of force is an important characteristic in the CMJ and may be a distinguishing performance factor in rowing performance.

## Limitations

The participants in this study had the opportunity to complete the CMJ at their convenience during the 2023 USACINC. Unfortunately, this did not allow the research team to control for timing of the participants' CMJ repetitions in relation to their indoor rowing competition, potentially influencing their performance through prior exposure to high-intensity exercise. As a result, some participants' CMJ performance may not be reflective of their true capabilities, especially if they performed the test in the first hour after their indoor rowing event. Additionally, familiarization of the testing with participants was limited. Finally, due to a small sample of female rowers their data was excluded. Future research should focus on exploring CMJ force-time characteristics in female rowers to see if these same relationships are present. Furthermore, when investigating neuromuscular fatigue CMJ testing should include baselines testing as well as testing during periods of high volume and/or intensity, and following this period to more clearly identify any potential changes (67).

## Conclusion

This study is the first to identify key CMJ variables with the strongest relationship to 2k erg performance. The findings highlight the importance of impulse in both the CMJ and rowing stroke. Future research should examine if these CMJ variables can be used as specific measurements of neuromuscular status in rowing athletes and to determine if this could be used as a way of talent identification. Furthermore, strength and conditioning coaches and rowers can use this information to help individualize training programs and exercise selection to improve neuromuscular function as a contributing factor to anaerobic performance, including the targeting of long SSC movements of the lower body. Finally, these relationships became stronger when controlling for previous or current rowing-related LBP.

## Data Availability

The raw data supporting the conclusions of this article will be made available by the authors, without undue reservation.
